# E-diagnostic assessment of collaborative and individual oral tiered task performance in differentiated second language instruction framework

**DOI:** 10.1186/s40468-023-00223-7

**Published:** 2023-02-02

**Authors:** Fahimeh Rafi, Natasha Pourdana

**Affiliations:** Department of Teaching English and Translation, Karaj Branch, Islamic Azad University, Moazen Boulevard, Gohardasht, Karaj, Alborz 31485-313 Iran

**Keywords:** Collaboration, Diagnostic assessment, Differentiated instruction, Google Meet, Tiered task

## Abstract

Differentiated second/foreign language (L2) instruction (DI) has received growing attention in pedagogical research. Yet, as a cornerstone of inclusive education, the DI is still under-documented. In this study, we integrated diagnostic assessment (DIA) and collaborative language learning in a differentiated (tiered) oral tasks intervention and mediated them in a Google Meet cyber classroom. To do so, an intact group of 64 non-English major university students was selected, pretested, and randomly assigned to collaborative (CG) and individual (IG) groups. Each group was partitioned into higher, middle, and lower language ability tiers. The objectives were to examine (1) how differently mixed-ability English-as-a-foreign-language (EFL) learners could improve their collaborative and individual oral tiered task performances on which they received e-DIA for 10 weeks, and (2) how differently e-DIA could affect their postintervention learning achievement in oral skills. The statistical analysis indicated the positive and equal impact of e-DIA on CG-tiers, but a positive and divisive impact on IG-tiers. Responded to e-DIA, both groups had significant pretest-to-postest learning achievement in oral skills, but CG-tiers’ collaboration resonated with the impact of e-DIA to outperform the IG-tiers on the posttest. The findings implied the integration of e-DIA and collaborative learning in the DI framework to mitigate L2 learners’ diversity in inclusive L2 education.

**Supplementary Information:**

The online version contains supplementary material available at 10.1186/s40468-023-00223-7.

## Introduction

Nowadays, one of the most difficult situations that second and foreign language (L2) teachers often face is the plurality in the student population in terms of their diverse language knowledge, motivation, giftedness, learning styles, willingness to participate, and level of maturity (Esfandiari & Noor, 2018; Nour et al., 2021; Pourdana et al., 2021; Rafi et al., 2022). Looking at this reality from a historical perspective, two main approaches are often resorted to by L2 teachers as an appropriate response. They prefer either to (1) select a homogeneous group of students from the mixed-ability student population to promote educational equity or to (2) accept the ad hoc levels of heterogeneity but incorporate differentiated instruction (hereafter, DI) to adjust their teaching to individual student’s level of academic readiness and the learning road map (Csapó & Molnár, 2019; Pourdana & Rad, 2017).

The former approach most likely fails as students are diverse in many ways and their differences grow fast over time. Therefore, reaching for mass-produced instruction based on “the template of grade-level expectations” would result in segregated education (Tomlinson et al., 2003, p. 120). Recently, the latter approach has received several initiatives as L2 practitioners began to delve into the demographic realities of the students, meet their individual needs, and maximize their successful learning in an inclusive educational setting (Finkelstein et al., 2019; Tomlinson et al., 2014).

Facing the challenge of a mixed-ability learning environment, L2 teachers are encouraged to use the DI framework to offer the students several avenues to move on from their current level of knowledge and skills to a new level of high-quality learning (Tavassoli et al., 2022). By definition, the DI is “a strategy that involves approaching every student as an individual. It is an alternative to a one-size-fits-all approach to education” (Ritter, 2018, p. 12). Parsons et al. (2018) promoted the DI as “a cornerstone of effective instruction” and “the gold standard teachers should strive for” (p. 206).

A hallmark of the DI practice is when the L2 teachers arrange two or more fluid instructional levels or *tiers* through which they differentiate the students based on their current proficiency levels for each instructional concept (Lindner & Schwab, 2020; Pourdana & Tavassoli, 2022). In this way, teachers can feed higher-ability learners with relatively complicated tasks, while the middle and lower-ability learners can be assigned the less challenging or easier versions of the same tasks. In other words, the tasks are tailored or *tiered* to the students’ cognitive capacities (Levy, 2008).

Several challenges can obstruct the proactive DI intervention and subsequent mixed-ability classroom configuration. One of the baffling situations that DI practitioners commonly experience is *how* to know *what* exactly a learner knows. A possible solution to this problem is to launch an extant diagnostic assessment (hereafter, DIA) to measure differentiated instruction. By the same token, timely and sufficient DIA is a catalyst for adjusting pedagogical tasks to a mixed-ability group of L2 learners. Therefore, through DIA the L2 teachers can differentiate (1) the *content* or what students have learned, (2) the *process* or how students have made sense of the instructions, and (3) the *product* or how students can demonstrate their target language use (Tomlinson et al., 2003). Yet, despite the growing interest in DIA as an alternative assessment that targets the diversity of L2 learners, it is still under-documented in response to differentiated instruction on oral communication skills (Alderson et al., 2015; Rafi et al., 2022).

Another enduring problem in DI pedagogy is created by the material costs, time pressure, and inadequate professional experience that impedes the systematic and ongoing DIA practice (Nunley, 2006; Pourdana & Mohammadi Zenouzagh, 2021). It becomes even worse when conventional teacher-directed instructions resist the constructionist student-centered approaches to collaborative language learning (hereafter, CLL) (Kazemi et al., 2022). CLL is viewed as mutual attempts by a pair/group of students to co-construct a message and convey it in the L2 (Ismail et al., 2019). As a didactic device, CLL not only welcomes plurality in L2 learners but also encourages L2 learners to co-build their knowledge of language via interactions. Also, CLL is an effective attempt to respond to the individual needs of L2 learners inside a classroom (Lindner & Schwab, 2020). It primes form-meaning negotiation opportunities as the learners are deeply engaged in processing the target language and reflecting upon it (Keshanchi et al., 2022). Despite its action potential, CLL has many unexploited trajectories in the L2 inclusive educational setting.

From a different pedagogical perspective, the advancement of computer-mediated communication (CMC) has intrigued many SLA researchers and educators to notice its potential in provoking a large amount of collaboration in L2 learning (Reid & Feist, 2018), classroom management (Kazemi et al., 2022), and effective uses of resources (Huang et al., 2021). More precisely, in the absence of face-to-face collaboration in distance or emergence education during the COVID-19 pandemic, CMC could play a critical role to promote collaborative agencies in L2 learning. However, only a few studies have examined the dynamic of CMC forums such as Google Meet™ or Zoom™ to deliver e-DIA (Pourdana, 2022) or integrate e-DIA and CLL in a mixed-ability classroom (Rafi et al., 2022).

To void the gap in the literature, therefore, this study used the DI framework to investigate (1) how e-DIA might affect the collaborative and individual performances of the mixed-ability EFL learners on oral tiered tasks and (2) whether e-DIA might improve learning oral skills by the mixed-ability EFL learners on Google Meet cyber classroom. This empirical study addressed the following research questions:

RQ1: Does the online diagnostic assessment have any impact on the *learning progress* of oral skills by mixed-ability EFL learners’ who performed the weekly oral tiered tasks collaboratively and individually? If yes, how?

RQ2: Does the online diagnostic assessment have any differential impact on the *learning achievement* of the mixed-ability EFL learners who performed oral tiered tasks collaboratively and individually? If yes, how?

## Literature review

### Differentiated instruction

Founded on Vygotsky’s (1987) sociocultural theory of mind (SCT), the DI model aims to make sure that every student has adequate access to a well-designed curriculum and the chance of becoming a successful learner. Moreover, the DI is viewed as the teachers’ integrated strategies to *include* those students who are cognitively and socioculturally diverse in the same classroom. L2 teachers can use the DI to (1) plan and adapt their teaching routines, contents, and tasks; (2) identify the student’s individual learning needs; and (3) equally maximize learning opportunities for individual students (Pham, 2012). The DI can anchor Vygotsky’s zone of proximal development (ZPD) in terms of a proactive set of instructional plans. They are built upon constant diagnostic feedback and scaffolding via the expert-novice interactions between the teacher and the students (Borja et al., 2015; Tavassoli et al., 2022).

Also, the DI provides multiple mediums for differentiating the content, the process, and the output/product of language learning (Tomlinson et al., 2014). By differentiating these curricular components, L2 teachers “offer different approaches to *what* students learn, *how* they learn it, and how they demonstrate what they’ve learned” (Tomlinson et al., 2003, p. 5). Firstly, differentiating the content can be thought of as adapting *what* the teachers teach or modifying *how they give students access* to the content. According to Tomlinson et al. (2003), content differentiation is “a response to the student readiness, interests, and learning profile” (p. 73). The teacher’s goal in readiness differentiation of the content is to match the content to the students’ cognitive capacity, while the interest differentiation of the content involves selecting materials based on the students’ current interests, and the learning profile differentiation of the content implies matching it to the students’ preferred learning styles such as using overhead transparencies in classroom discussions to link the visual and auditory learning.

Secondly, as an essential component of the DI, *the process* is a sense-making activity that is mapped to the language content to which L2 learners are exposed (Suwastini et al., 2021). Process differentiation also corresponds to the readiness, interest, and learning profile of L2 learners. Readiness differentiation of the process requires matching or *tiering* the task complexity to L2 learners’ current level of understanding, while the interest differentiation of the process involves helping students to link their interests to a sense-making goal (Bahador & Mofrad, 2020). Lastly, the learning profile differentiation of the process is viewed as encouraging L2 learners to make sense of a concept in their preferred learning style, for example, expressing their thoughts kinesthetically or verbally (Tomlinson et al., 2003).

Finally, *output/product* differentiation can help L2 learners to demonstrate their learning achievement in various modes (Pham, 2012). As Heacox (2012) added, language “output reflects what students have understood and been able to apply. They show learning in use and may reveal new thinking or ideas” (p. 11). Therefore, a top-rate output task can cause L2 learners to think, apply, and stretch their knowledge in pursuit of quality. The output-oriented approach to the DI is based on the linear and regular L2 teacher’s diagnostic assessment of the students (Chen, 2007).

One of the unique representations of differentiated output is the tiered task, which fosters the interaction between assessment and instruction. Tiered tasks involve “teaching similar language contents to the whole class, but assessing individual students with graded tasks well-suited to their cognitive capacities” (Rafi et al., 2022, p. 3). On tiered tasks, L2 learners can collaborate or work individually, while the tier membership is carefully assigned by the teacher based on the students’ level of readiness or academic achievement (Levy, 2008). Before designing tiered tasks in the DI classroom, L2 learners are stratified or graded based on a pre-assessment. Those in Tier 1 are known as the higher-ability students whose level of language proficiency goes beyond the norm of the class. Tier 2 includes middle-ability students whose level of readiness usually represents the de facto norm of the class. Finally, in Tier 3, the L2 learners are known as the lower ability who can only adapt to the norms with the teacher’s scaffolding (Pourdana & Rad, 2017).

### Diagnostic assessment in differentiated instruction

In a DI classroom, the L2 teacher does not assume that one size fits all in assessing students. As a result, timely and regular DIA is inseparable from the DI instructions. Accordingly, L2 teachers are required to monitor the individual students and “identify their instructional needs by analyzing assessment data, evaluating student progress, and setting challenging goals based on the curriculum and students’ needs” (van Geel et al., 2019, p. 53). Therefore, the DI proponents believe that DIA is *educative* and a prerequisite in the DI classroom which establishes “rich qualitative data about the individual’s cognitive abilities, psychological pathologies, and personalities” (Gorin, 2007, p. 7).

Diagnosis is denoted as the “investigation or analysis of the cause or nature of a condition, situation, or problem” (Diagnosis, 2022). The three major benefits of DIA in a mixed-ability classroom were counted by Alderson et al. (2015) as (a) being personalized and learner-centered, (b) making an explicit focus on the remedies (therapies) in future performance, and (c) providing deep analysis of the challenging areas that need attention. In the same vein, Harding et al. (2015) operationalized the stages of DIA as follows:

1. Observing the individual participants’ weaknesses and strengths
2. Providing explicit directions to address the problematic area
3. Suggesting better and more effective learning plans and appreciating the strong points in the students’ performance
4. Providing step-wise, individualized, and detailed diagnostic feedback
5. Making summative decisions

The notion of “step-wise DIA” in a mixed-ability classroom is highly relevant to the assessment of oral language skills at the level of letter-sound correspondence (pronunciation) (Rafi et al., 2022). According to Atkinson (2018), the language sound articulation benefits from the lower-level and higher-level processes. Lower-level processes enable L2 users “to recognize the sounds, lexical segments, and syntactic parsing, while the higher-level processes involve the (meta) cognitive strategies, inferences, and monitoring intelligibility” (Rafi et al., 2022, p. 5).

The complex processes of sound production are incorporated into the psychomotor domain of human learning. The psychomotor domain accounts for the progressive levels of learning behaviors from the less complex (e.g., imitation) to the more complex (e.g., natural sound articulation) (Atkinson, 2018). Therefore, psychomotor skills demand physical or tactile components. In other words, “rather than using the mind to think (cognition) and reflect (metacognition), or even the ability to develop social skills (affect), psychomotor behaviors are the things we do physically” (Rafi et al., 2022, p. 5). As a result, they need high levels of flexibility, delivery, coordination, and motor control.

In SLA research on L2 learners’ clinical skills, Atkinson’s (2018) Hierarchy of Intended Learning Outcomes (ILOs) is one of the most referenced taxonomies (Table 1). In his hierarchy, the psychomotor descriptors are used to introduce transferrable practical skills, indicate how the primary skills should move towards ILOs, and usher the L2 teachers’ DIA of the learners’ learning progress in pronunciation.

**Table 1:** The psychomotor domain taxonomy of oral skills (adapted from Atkinson, 2018)

Stage	Proto-verb	Descriptor
Imitation	(to) imitate	The ability to observe, copy, and replicate the speech sounds of others
Manipulation	(to) manipulate	The ability to articulate sounds by memory or repeat/reproduce speech to prescribed standard instructions
Precision	(to) perfect	The ability to articulate speech sounds without interventions from others, with a high degree of accuracy and few errors
Articulation	(to) articulate	The ability to adapt, assimilate or integrate existing psychomotor skills in a novel and non-standard way (e.g., a dialect or accent)
Naturalization	(to) embody	The ability to articulate natural or near-natural speech in an automatic, intuitive, or unconscious way appropriate to the context

By adopting the ILO taxonomy into the L2 letter-sound mapping, oral language skills proceed from the primitive verbal behavior (i.e., imitation) through the complex sound production skill (i.e., naturalization). In other words, in mastering the intended quality of target sounds pronunciation, imitating the native speaker’s articulation seems the least demanding, and producing fluent native-like sounds is the most demanding psychomotor skill. Because one of the key attributes of DIA is the step-wise assessment of tiered task performance, Atkinson’s (2018) ILOs could suitably serve as a theoretical framework to tier the oral language tasks in the current study.

### Communication technologies in differentiated instruction

Recently, with the surge of the COVID-19 pandemic, CMC technologies have transformed teaching, learning, and language use on daily basis. The CMC and information technologies (IT) also have the potential to promote the efficiency of DIA and the subsequent student decision-making in a mixed-ability classroom (Alderson et al., 2015). Unlike the conventional assessment practice by L2 teachers, computerized diagnosis hardly tires the students as it provides synchronous and automated corrective feedback in a less obtrusive and more user-friendly way (Ritter, 2018).

CMC can actively contribute to differentiating the content, process, and output/product of the L2 learning process (Shepherd & Alpert, 2015). E-differentiation *by content* can be accomplished by (a) adopting and digitalizing content to teach the same material to diverse students with various needs and (b) enhancing the content to make it accessible to all learners in terms of digital books, simulations, visualizations, applications, etc. (Karatza, 2019). E-differentiation *by process* works effectively as L2 teachers can incorporate CMC forums to provide the students with several options for exploring, researching, studying, and evaluating both teaching concepts and learning objectives (Taylor, 2015). The IT systems can differentiate not only the processing, manipulating, and recording of information but also the time individual students require to do the task (Scalise, 2007). E-Differentiation *by output/product* enables each student to demonstrate their learning by utilizing Web 2.0 technologies such as Podcasts, Blogs, Wikis, digital narrative applications, shared/collaborative presentations (e.g., Prezi), etc.

The CMC literature on improving accuracy in pronunciation (Seferoglu, 2005), the L1 to L2 transfer of sounds articulation (Wang & Munro, 2004), and achieving native-like stress and intonation patterns (Levis & Pickering, 2004) supported the facilitating role of advanced technologies in learning oral skills in the target language. Among the CMC platforms in L2 pedagogy, Google Meet (formerly *Hangouts*) is a popular and free multimedia communication service. Google Meet allows around 100 attendees to join a virtual meeting and have real-time interactions. As a pedagogical CMC platform, Google Meet can easily generate communicative learning environments for diverse group sizes, such as the whole class, small groups, and dyads (Rafi et al., 2022).

### Collaboration in differentiated instruction

Vygotsky (1987) highlighted the sequential link between collaborative language learning (CLL) and independent task performance by predicting what L2 learners can do collaboratively today and what they can do unmediated tomorrow. CLL in the DI pedagogical practice has recently attracted the attention of more educators and L2 teachers since both concepts are used to adapt instructions to students’ diverse levels of readiness and language achievement (Ismail & Al Allaq, 2019).

Through collaboration, “L2 learners no longer need to rely exclusively on their L2 knowledge resources when performing a challenging task” (Rafi et al., 2022, p. 5). In other words, they can have a substantive body of interactions in different stages of the L2 learning process for collecting mediation from the teacher and the peers (Pourdana & Asghari, 2021; Storch, 2013). Such collaborative scaffolding can be seen in negative feedback (error correction), positive feedback (approval), or diagnostic feedback (remedial) (Donato, 1994). They also can share their plans and equally feel responsible for their mutual task outcome. Therefore, the integration of CLL and DIA practice might strengthen the teacher-student and student-student synchronous interactions in the CMC educational outlets (van Geel et al., 2019).

The integral role that DIA and CLL can play together in the DI might be amplified by using advanced technologies on CMC trajectories. Exploring this synergy was an untaken road this study planned to endeavor. Therefore, we intended to investigate the potential impact of e-DIA and CLL embedded in the DI on mixed-ability EFL learners in terms of their weekly progress on oral tiered task performance and their L2 learning achievement. The target audience in this study was the L2/EFL teachers, material and assessment task developers, and SLA researchers.

## Method

### Context and participants

This experiment was carried out at a large university campus in Iran during the COVID-19 pandemic. An intact group of 64 Persian-speaking EFL learners (35 females, 54.69%) who were freshman university students of Psychology took part in this study whose participation in the 10-week online sessions was a partial fulfillment of the General English course requirements. Their ages ranged from 18 to 25 (*M* = 21.08, *SD* = .71), and their formal exposure to English was about 12.3 years on average.

The criteria for participating in this research were easily met by all students, including owning a smartphone or a desktop computer, having regular access to the Internet or WiFi network service, and registering to Google Meet.

Participants’ levels of English oral proficiency were measured by administering the Preliminary English Test (PET): Listening Sample Paper 1 (UCLES, 2004). The 25 items of the PET were converted into Google Forms™, a free survey administration software. The participants were required to complete the online version of the PET in 35 min by downloading and listening to the audio file attached to the Google Forms (Cronbach’s *α* = .882). Before the intervention, the participants were assigned to the collaborative (CG, *N* = 34) and individual (IG, *N* = 30) groups, each further divided into the higher, middle, and lower ability *tiers* (divisions) based on their obtained PET scores (Table 2).

**Table 2:** Demographic information of the participants

Group	Proficiency level	PET score range	Tier	*N*	Studying English (year)
CG	Higher ability	21–25	1	5 dyads	> 14
	Middle ability	17–20	2	6 dyads	11–14
	Lower ability	10–16	3	6 dyads	9–11
IG	Higher ability	21–25	1	10	> 14
	Middle ability	17–20	2	12	11–14
	Lower ability	10–16	3	8	9–11

After grouping, and tier assignment, the PET scores were compared across the tiers in the CG and IG and no between-group statistical differences were found, respectively [*F* (2, 31) = .678, *p* = .72, partial, *η*^*2*^ = .003; *F* (2, 27) = .478, *p* = .65, partial *η*^*2*^ = .009]. During the treatment sessions, the participants remained in their assigned tiers.

### Instruments and materials

#### Preliminary English Test (PET)

Preliminary English Test (PET): Listening sample paper 1 consists of 7 multiple-choice picture-cued items, 6 multiple-choice listening comprehension items, 6 open-ended listening comprehension items, and 6 true-false listening comprehension items (*N* = 25). The responses are measured dichotomously (1 for a correct response, 0 for an incorrect response). In this study, participants’ English oral proficiency level was measured with the electronic version of the PET, as the placement and pretest in 35 min. As the posttest, another electronic version of the PET (listening sample paper 2) was run a week after the 10-week treatment sessions.

#### Oral tiered tasks

A pool of 130 English words was selected from the assigned General English coursebook *Select Readings: Intermediate* (Lee et al., 2011). This sampling was conducted based on the 91.30% index of familiarity, obtained from a pilot group of students (*N* = 27), and the researchers’ record of English words with confusing/difficult pronunciation to Persian-speaking EFL learners. On this account, 100 words were selected and incorporated into 10 sets of oral-tiered tasks.

Next, a body of 30 oral-tiered tasks (*N* = 3 tasks ×10 sessions) was developed by the researchers after adopting Atkinson’s (2018) ILO taxonomy as the theoretical framework. The researcher-made oral tasks were tiered based on their (1) language modality (i.e., recognition, recognition-production, production) and (2) intended learning outcome (ILOs) (i.e., imitation, manipulation, precision, articulation, and naturalization) (Table 3).

**Table 3:** Modality and ILOs of the oral tiered tasks

Tier modality		ILOs				
		Imitation	Manipulation	Precision	Articulation	Naturalization
1	Production	**-**	**-**	**-**	**-**	**+**
2	Recognition + production	**-**	**-**	**+**	**+**	**-**
3	Recognition	**+**	**+**	**-**	**-**	**-**

Accordingly, the tasks in Tier 1 were operationalized based on *naturalization* ILO in the *production* modality; the tasks in Tier 2 were developed based on *precision* and *articulation* ILOs in the integrated modalities of *recognition + production*, and the tasks in Tier 3 were generated based on *imitation* and *manipulation* ILOs in the *recognition* modality. The two-dimensional attributes of the tiered tasks enabled the researchers to grade them as complex (Tier 1), moderate (Tier 2), and easy (Tier 3).

The developed tiered tasks were parallel in their content for both the CG and IG. For instance, when the higher-ability students in Tier 1 were required to pronounce the word *reactionary* aloud (i.e., production/naturalization), the middle-ability students in Tier 2 had to pronounce *reactionary* after listening to an English native speaker and adjust their pronunciation (i.e., recognition + production/precision + articulation), and the lower ability students in Tier 3 had to listen to the recorded pronunciation of *reactionary* by a non-native English speaker and recognize whether it was pronounced accurately (i.e., recognition/imitation) (see Additional file 1: Appendix A for a sample of tiered tasks).

The content validation of the developed tiered tasks was performed by four university professors majoring in Teaching English as a Foreign Language (TEFL) and language assessment who reviewed and rated each task based on the criteria of appropriateness, clarity of the task rubric, length of tasks, and level of complexity. The disagreements in the rating procedure were resolved to reach a full consensus. The revised tiered performance tasks were piloted with 27 undergraduate students similar to the participants of the study (Cronbach’s *α* = .829).

#### Data collection procedure

**Fig. 1:**
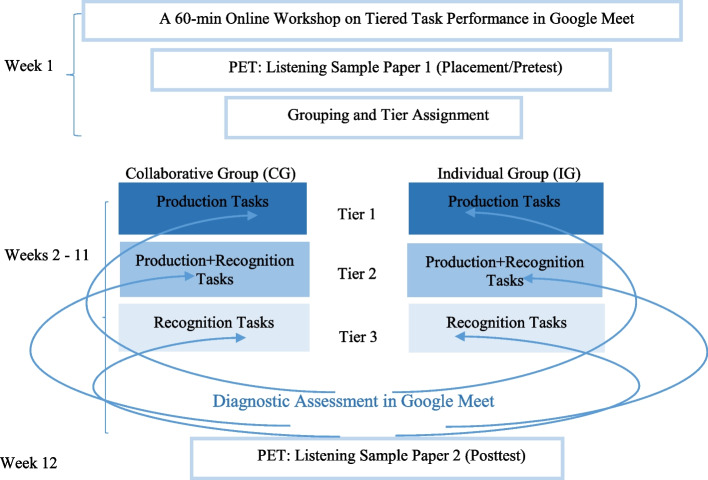
The procedure

The flow of the data collection procedure is illustrated in Fig. 1. An intact group of 64 non-English major EFL university students was selected through the convenience sampling method. In Week 1, in a 60-min online workshop, the researchers introduced the participants to the structure of tiered tasks, shared their learning goals and success criteria, and screen-shared a sample of oral tiered tasks on Google Meet.

After signing a consent form and partaking in a digital version of PET: Listening sample paper 1 in Google Forms, the participants were randomly assigned to collaborative and individual groups. Next, based on their PET scores, the CG and IG members were partitioned into three disguised tiers of the higher, middle, and lower-ability students.

In the next 10 weekly sessions, the time of the online classes was divided into 45 min of regular English instructions, and 45 min of the performance on the oral-tiered tasks and subsequent DIA. In both groups, the task input was presented to the tiers separately but simultaneously in Google Meet. In 20 min, the CG-tiers completed a given task collaboratively, while the IG-tiers were required to perform it solitarily. The researchers were able to randomly monitor all participants through Gallery View in Google Meet.

For the next 25 min, the researchers hosted parallel Google Meet sessions to provide collective diagnostic feedback on committed errors in the previous session’s tiered task output. The common errors were given priority to receive more time, focus, and choral/individual practice. The researchers coached the participants with effective plans and strategies for learning accurate pronunciations such as using *Titan* or *Say It* speech analyzing software and online dictionaries. After the treatment sessions, the participants in both CG and IG individually took part in another virtual version of PET (listening sample paper 2) as the posttest (Cronbach’s *α* = .812).

#### Data analysis

The objective of the first research question was to investigate whether e-DIA might differentially impact the weekly performance of the higher ability, middle ability, and lower ability tiers in the CG and IG on Task 1 to Task 10. Any significant improvement in the CG-tiers and IG-tiers’ task performance (dependent variable) was interpreted as their *learning progress* in oral skills due to receiving e-DIA (independent variable) over the 10 weeks of intervention. Research question 1 was examined by running the mixed between-within-subject analysis of variance (ANOVA) (Tabachnick & Fidell, 2013) for the CG-tiers and IG-tiers, separately.

The second research question investigated whether the collaborative and individual dynamics of tiered task performance (moderator variable) could modify the impact of e-DIA on learning oral skills by the CG and IG. Any observed outperformance of the groups on the posttest was interpreted as their *learning achievement*. Moreover, the significant differences between the pretest-to-posttest gain scores of the CG and IG were interpreted as an interaction between group membership and the e-DIA effect. Research question 2 was examined by running a one-way analysis of covariance (ANCOVA) to control for the possible effect of the pretest as the covariate (Pallant, 2011).

## Results

Initially, the normality assumption of the obtained data out of the tiered task scores, the pretests, and the posttests for the CG and IG was examined. According to the normality reports, the ratios of skewness and kurtosis over their standard errors for tiered tasks, the pretest, and the posttest scores of the CG and IG were within the range of ± 1.96 to indicate that the collected data were normal (Byrne, 2010). Next, descriptive statistical analysis was conducted (Table 4).

**Table 4:** Descriptive statistics: tiers in collaborative and individual groups

Group	Tier	Mean	SD	Std error	95% Confidence interval	
					Lower bound	Upper bound
Collaborative	1	6.290	.204	.103	6.205	6.797
	2	6.101	.158	.103	5.901	6.720
	3	5.890	.109	.103	5.110	6.341
Pretest		17.201	.401	.103	16.390	17.871
Posttest		22.109	.391	.103	21.501	22.650
Individual	1	6.790	.701	.201	5.991	6.841
	2	5.724	.540	.201	5.114	6.310
	3	5.401	.401	.201	6.093	6.983
Pretest		17.117	.440	.201	16.981	17.541
Posttest		21.761	.320	.201	21.117	22.540

As Table 4 reports, some similarities and differences existed between the tiered task performances of the CG and IG. Accordingly, in both groups, Tier 1 outperformed Tier 2 and Tier 3 throughout the 10-week task performance. Moreover, both groups improved their performance from the pretest to the posttest. Yet, the CG outperformed the IG on the posttest (*M* = 22.109 ± .391, *M* = 21.761 ± .320, respectively).

To further examine the observed differences across CG-tiers and IG-tiers, the mixed between-within-subjects ANOVA—an extension of the repeated measures designs (Tabachnick & Fidell, 2013)—was run. Three major assumptions to be checked in this statistical analysis were (1) Levene’s test of equality of error variances, (2) the equality of covariance matrices, and (3) the sphericity. The results of Levene’s test measured for CG-tiers and IG-tiers’ performance across Tasks 1 to 10 were all above the critical value (*p* > .05), so they did not violate the assumption of homogeneity of variances. The results of Box’s M test for the CG and IG (*M* = 14.197, *p* = .350 > .001, and *M* = 13.107, *p* = .210 > .001, respectively) indicated that this assumption was also retained. Finally, for the CG and IG, the results of Mauchly’s test of sphericity (*χ*^2^ (2) = .946, *p* = .158 > .05 and χ^2^ (2) = 1.046, *p* = .274 > .05, respectively) indicated that the assumption of sphericity was met in both groups.

### Impact of e-DIA on CG-tiers’ task performance

Before investigating the effect of e-DIA on the CG-tiers’ weekly task performance, the presence of any interaction effect was assessed (Table 5).

**Table 5:** Multivariate tests of within-subject effect in the collaborative group

Source		Value	*F*	Hypothesis df	Error df	Sig.	Partial *η*^2^
Time	Pillai’s trace	.906	73.94	7.00	22.00	.000	.906
	Wilk’s Lambda	.094	73.94	7.00	22.00	.000	.906
	Hotelling’s trace	9.585	73.94	7.00	22.00	.000	.906
	Roy’s largest root	9.585	73.94	7.00	22.00	.000	.906
Time * tiers	Pillai’s trace	.336	5.763	7.00	46.00	.060	.018
	Wilk’s Lambda	.328	5.763	7.00	44.00	.060	.018
	Hotelling’s trace	.358	5.763	7.00	44.00	.060	.018
	Roy’s largest root	.247	5.763	2.00	23.00	.060	.017

According to Table 5, the measure of Wilk’s lambda for Time * Tiers (*Λ* = 5.763, *F* (7, 44) = .328, *p* = .060, partial *η*^*2*^ = .018, interpreted as a weak effect size) (Lenhard & Lenhard, 2016) indicated that the interaction effect was insignificant. In other words, the collaborating participants in the CG-tiers equally benefitted from the impact of e-DIA on their task performance. Moreover, the measure of Wilk’s lambda for the main effect of time was significant (*Λ* = 5.763, *F* (7, 22) = 73.94, *p* = .000, partial *η*^*2*^ = .906, interpreted as a very large effect size) and indicated that there was a noticeable internal change (i.e., learning progress) in the performance of the CG-tiers from Task 1 to Task 10. To explore any differences across the CG-tiers, the between-subject effect was tested.

As Table 6 shows, no significant between-subject effect was found in the CG-tiers, *F* (2, 32) = 14.03, *p* = .090, partial *η*^*2*^ = .019, representing a weak effect size). In other words, no distance or diversity was observed across the CG-tiers who received e-DIA on their collaborative tiered tasks performance.

**Table 6:** Test of between-subject effects in collaborative group

Source	Type III sum of squares	df	Mean square	*F*	Sig.	Partial *η*^2^
Intercept	127454.525	1	127464.525	1099.008	.000	.994
Tiers	61.946	2	30.973	14.033	.090	.019
Error	132.429	32	2.207			

### Impact of e-DIA on IG-tiers’ task performance

Similar procedures were undertaken in the analysis of the oral-tiered task performance by the IG-tiers over 10 weeks. According to Table 7, the measure of Wilk’s lambda for Time * Tiers (*Λ* = .328, *F* (14, 36) = .328, *p* = .000, partial *η*^*2*^ = .428, interpreted as a large effect size) indicated that a significant interaction effect existed among the IG-tiers. In other words, the individual participants reacted differently to the impact of e-DIA on their weekly task performance.

**Table 7:** Multivariate tests of within-subjects effect in individual group

Source		Value	*F*	Hypothesis df	Error df	Sig.	Partial *η*^2^
Time	Pillai’s trace	.406	80.04	7.00	18.00	.000	.703
	Wilk’s Lambda	.494	80.04	7.00	18.00	.000	.703
	Hotelling’s trace	7.505	80.04	7.00	18.00	.000	.703
	Roy’s largest root	7.505	80.04	7.00	18.00	.000	.703
Time * tiers	Pillai’s trace	.736	4.574	14.00	36.00	.000	.368
	Wilk’s Lambda	.328	5.763	14.00	36.00	.000	.428
	Hotelling’s trace	1.858	7.035	14.00	36.00	.000	.482
	Roy’s largest root	1.747	13.730	7.000	18.00	.000	.636

Moreover, the measure of Wilk’s lambda for the main effect of time was significant (*Λ* = .494, *F* (7, 18) = 80.04, *p* = .000, partial *η*^*2*^ = .703, interpreted as a large effect size). In other words, similar to the CG-tiers, meaningful progress in the tiered task performance of the IG-tiers could be observed from Task 1 to Task 10. The differences across IG-tiers were examined with the test of the between-subject effect.

As Table 8 indicates, the between-subject effect was significant across the IG-tiers, *F* (2, 28) = 10.057, *p* = .000, partial *η*^*2*^ = .319, interpreted as a large effect size. In other words, in the IG, the tiers grew apart and remained diverse as they solitarily completed Task 1 to Task 10.

**Table 8:** Multivariate tests of within-subjects effect in individual group

Source	Type III sum of squares	df	Mean square	*F*	Sig.	Partial *η*^2^
Intercept	23,393.525	1	23,393.525	10,599.008	.000	.994
Tiers	41.906	2	41.903	10.057	.000	.319
Error	132.429	28	2.207			

To sum up, the statistical analysis indicated that the CG and IG responded differently to the e-DIA in the Google Meet cyber classroom. The CG-tiers had considerable progress in their oral task performance and e-DIA positively and equally affected them. So did the IG-tiers whose progress in tiered task performance was significant due to e-DIA. Yet, as a point of departure, the diversity among the IG-tiers resonated after receiving e-DIA on their oral tiered task performance.

### Impact of e-DIA on learning oral skills by collaborative and individual groups

The second research question addressed the impact of e-DIA on learning oral skills by the CG and IG in terms of their pretest-to-posttest learning achievement. A one-way analysis of covariance (ANCOVA) was run with the mean scores of the two groups on the posttest and their pretest mean scores as the covariate. The assumptions of ANCOVA were met in this study, including the test of normality (i.e., indicated by the desirable ratios of skewness and kurtosis), and the homogeneity of variances measured by Levene’s test of equality of variances (*F* (1, 60) = .241, *p* = .867).

The results of ANCOVA indicated the main effect of e-DIA which caused a significant difference between the mean scores of the CG and IG on the posttests (*F* (1, 60) = 11.120, *p* = .000, partial *η*^*2*^ = .063, interpreted as a medium effect size). Referring to Table 4, the outperformance of the CG on the posttest had to be interpreted as significant (*M* = 22.109, *SD* = .391, 95% CI [21.501—22.650]).

## Discussion

The discussion of the first research question is two-fold. Firstly, the positive impact of DIA on the CG-tiers and IG-tiers who completed the tiered tasks collaboratively and solitarily can be discussed from the Vygotskyan (1987) constructivism perspective. Accordingly, the “cohort impact of differentiated instruction and the follow-up diagnostic assessment” is deemed to scaffold the cognitive capacities of the L2 learners with various levels of readiness and frames of reference” (Rafi et al., 2022, p. 14). In other words, the knowledge of L2 is actively constructed within the limits and offerings of the learning environment.

Gorin (2007) and Lee (2015) attributed DIA closely to the potential of the immediate *penetration*, fine-grained, and tailored assessment that pinpoints the root causes of the mixed-ability students’ challenges within a class community. In the DIA environment, through the expert’s (teacher or peer) ongoing observation and individualized error description, the students are collectively scaffolded and approach their *zone of proximal development* (Abdulaal et al., 2022). By the same token, DIA can provide L2 learners with adequate explicit knowledge to self-monitor their task performance and improve their task product/output.

Secondly, the mixed-ability students whose collaborative tiered task performance received e-DIA benefitted more than those who completed the weekly tiered tasks individually which eventually cost them more diversity and distance. This finding also anchors the constructive role of collaborative language learning (CLL) in Vygotsky’s SCT model (1987). SCT accentuates the double impacts of CLL and DIA in the L2 learning progress when collaborating students are pitched to their real cognitive capacities in a non-threatening environment (Yan-hong, 2013).

CLL can generate a sense of mutuality, rapport, and homogeneity (Storch, 2013). Through collaboration, the L2 learners “recognize a hole in their linguistic knowledge, and formulate and test their hypotheses about how the target language works, and in doing so, they co-build the knowledge that is new to them” (Swain, 2001, p. 99). In the differentiated instructional context, CLL is “a catalyst for academic and social improvement through a context of shared targets and welcoming learning environment” (Ismail & Al Allaq, 2019, p. 5). Also, CLL provides a sense of mutual accomplishment when L2 learners contribute to their peers’ learning within a tier, which in turn enhances their cognitive activity and positive attitude toward the target language (Gillies, 2004; Pourdana & Mohamadi Zenouzagh, 2021).

In the SLA literature, a few experimental studies investigated the importance of DIA and CLL in the DI context. Our findings on the first research question corroborate those of Rafi et al. (2022) and Ardin (2018). Rafi et al. (2022) focused on the impact of e-DIA on a diverse group of 66 Iranian EFL learners’ oral skill improvement. In a pretest-posttest experiment, the researchers reported noticeable improvement in the participants’ oral skills and their performance on post-intervention summative assessment. They also expressed strong approval of differentiated instructions, task-wise diagnostic assessment, and the Google Meet platform. Similarly, Ardin (2018) explored the positive effects of DIA on 40 EFL learners collaborating on descriptive and narrative writing tasks and reported a large effect size of DIA on both genres of academic writing.

The findings of the study are in partial contrast to Pourdana and Rad (2017) and Tozcu (2016). In a case study with 46 mixed-ability EFL learners, Pourdana and Rad (2017) reported the effectiveness of differentiated instructions in academic reading tasks. Yet, they could hardly track any direct association between the participants’ diverse language abilities and their reading task outcomes. Their unexpected results could be due to the flexible tier membership so the students had to move to the lower tier if they underperformed which most likely polluted the collected data in the study.

Tozcu (2016) investigated the impact of DIA on learning Turkish discourse markers by 24 military students in an intensive language program who were split into control and treatment groups. Participants’ performance on oral narrative tasks received regular DIA in the treatment group. Tozcu reported moderate improvement in only their production of grammatical sentence structures and cohesive devices. A possible argument for the limited impact of DIA in this study could be methodological and due to the small size of the participants in the treatment group.

On the second research question, the findings supported (1) the positive and enduring impact of e-DIA on learning oral skills in both the CG and IG and (2) the CG’s better record of learning achievement. The superiority of the CG can be argued in favor of the consequential validity of the DIA and the salient role of CLL (Lee, 2015) in empowering L2 learners when their diverse needs are respected in the DI classroom.

Likewise, Ellis (2012) promoted CLL as the integral component of an ideal inclusive educational setting where “the instructions are individualized which result in more negotiation of content, a greater variety of language use in initiating discussion, asking for clarification, and joking” (p. 815).

## Conclusion

In this study, we investigated how e-DIA could differentiate the 64 mixed-ability EFL learners’ collaborative and individual performances on the oral tiered tasks and manipulate their learning achievement of oral skills. Moreover, we examined how e-DIA could mitigate the diversity in those participants who performed tiered tasks collaboratively. Finally, we interpreted the posttest gain scores in both groups as the main effect of the e-DIA on their learning achievement and the outperformance of the collaborative group as the moderating role of collaboration.

The findings of this study provided strong evidence that integrating the DIA and collaboration in the DI practice can closely meet the attributes of inclusive L2 education. The broad sense of inclusive education identifies its five major aspects: (1) collaboration and teamwork; (2) instructional practices; (3) organizational practices; (4) social, emotional, and behavioral practices; and (5) determining progress (Finkelstein et al., 2019).

To adapt the inclusive education model to L2 learning pedagogy, *collaboration and teamwork* can involve mediation by the expert (the teacher or the more knowledgeable peer) and negotiation with the novice (the less knowledgeable peer). In *instructional practice*, L2 teachers can regularly generate the target language content and differentiate it to the levels of the students. *Organizational practice* demands L2 teachers to differentiate (meta) cognitive strategies and diversify the setup of the cyber or real classroom to stimulate individuals. *Social*, *emotional*, and *behavioral practices* require the L2 teachers to engage the mixed-ability students socially and emotionally and carefully attend to their behavioral needs (e.g., by encouraging collaboration and negotiation). Finally, *determining progress* involves individualized and formative assessment alternatives such as DIA to monitor the students’ task performance and learning outcomes. Undoubtedly, this tentative pedagogical framework requires further research and incorporation of its core concepts into inclusive L2 education.

The arguments in this research are not devoid of limitations. Firstly, the participants of the study were not screened for their demographic responses to the e-DIA, nor were their diversifying attributes such as gender, age, or multiculturality controlled by including comparison groups. The second major restriction was the COVID-19 pandemic and the irregularities and rearrangements it imposed on the course of data collection, data analysis, and follow-up discussions. Finally, from the research methodological perspective, this study was not planned to examine the sustainable impact made by e-DIA and/or CLL on the tiers’ learning oral skills by running the delayed posttests which is a challenging topic for further research.

## Supplementary Information

**Additional file 1:** **Appendix A.** Oral-tiered tasks # 4 (sample).

## Data Availability

Please contact the authors for data requests.

## Abbreviations

ANCOVA: Analysis of covariance
ANOVA: Analysis of variance
CLL: Collaborative language learning
CG: Collaborative group
CMC: Computer-mediated communication
DI: Differentiated instruction
e-DIA: Electronic Diagnostic Assessment
EFL: English as a Foreign Language
IG: Individual group
PET: Preliminary English test
L2: Second/foreign language
SCT: Sociocultural theory
ZPD: Zone of proximal development
