# Supplementary material for: E-diagnostic assessment of collaborative and individual oral tiered task performance in differentiated second language instruction framework
Source: Lang Test Asia. 2023 Feb 2;13(1):6. doi: 10.1186/s40468-023-00223-7 (PMC9892680; doi:10.1186/s40468-023-00223-7)

Borja, L. A., Soto, S. T., & Sanchez, T. X. (2015). Differentiating instruction for EFL learners. *International Journal of Humanities and Social Science, 5*(8), 30-36.

Byrne, B. M. (2010). *Structural equation modeling with AMOS: Basic concepts, applications, and programming (2^nd^ ed.).* Routledge Taylor & Francis Group.

Chen, Y.-u. H. (2007). *Exploring the assessment aspect of differentiated instruction: College EFL learners' perspectives on tiered performance tasks.* University of New Orleans University of New Orleans Theses and Dissertations. 837.

Csapó, B., & Molnár, G. (2019). Online diagnostic assessment in support of personalized teaching and learning: The eDia system. *Frontiers in Psychology, 10*, 1522. https://doi.org/10.3389/fpsyg.2019.01522

Diagnosis. (2022). In *The Merriam-Webster Online Dictionary*. Retrieved March 9, 2022, from www.merriam-webster.com/dictionary/diagnosis.

Donato, R. (1994). Collective scaffolding in second language learning. In J. P. Lantolf & G. Appel (Eds.), *Vygotskian approaches to second language research* (pp. 33-56). Ablex.

Ellis, R. (2012). *The study of second language acquisition (2^nd^ ed.)*. Oxford University Press.

Esfandiari, R., & Noor, P. (2018). Iranian EFL Raters’ Cognitive Processes in Rating IELTS Speaking Tasks: The Effect of Expertise. *Journal of Modern Research in English Language Studies*, *5*(2), 41-76. https://doi.org/10.30479/jmrels.2019.9383.1248

Finkelstein, S., Sharma, U., & Furlonger, B. (2019). The inclusive practices of classroom teachers: A scoping review and thematic analysis. I*nternational Journal of Inclusive Education*, 1–28. https://doi.org/10.1080/13603116.2019.1572232.

Gillies, R. M. (2004). The effects of cooperative learning on junior high school students during small group learning. *Learning and Instruction, 14*, 197-213. https://doi.org/10.1016/S0959-4752(03)00068-9

Gorin, J. S. (2007). Test Construction and diagnostic testing. In J. Leighton & M. Gierl, *Cognitive diagnostic assessment for education: Theory and applications*, (pp. 173 - 201). Cambridge University Press.

Harding, L., Alderson, C. J., & Brunfaut, T. (2015). Diagnostic assessment of reading and listening in a second or foreign language: Elaborating on diagnostic principles. *Language Testing,* 1–20. https://doi.org/10.1177/0265532214564505

Heacox, D. (2012). *Differentiating instruction in the regular classroom.* Minneapolis, MN: Free Spirit Publishing.

Huang, R., Tlili, A., Wang, H., Shi, Y., Bonk, C.J., Yang, J., & Burgos, D. (2021). Emergence of the online-merge-offline (OMO) learning wave in the post-COVID-19 era: A pilot study. *Sustainability*, *13*, 3512. https://doi.org/10.3390/su13063512

Ismail, S. A. A., & Al Allaq, K. (2019). The nature of cooperative learning and differentiated instruction practices in English classes. *SAGE Open,* 1-17. https://doi.org/0.1177/2158244019856

Karatza, Z. (2019). Information and communication technology (ICT) as a tool of differentiated instruction: An informative intervention and a comparative study on educators’ views and extent of ICT use. *International Journal of Information and Education Technology, 9*(1), 8-15. https://doi.org/10.18178/ijiet.2019.9.1.1165

Kazemi, P., Pourdana, N., Famil Khalili, F., & Nour, P. (2022). Microgenetic analysis of written languaging attributes on form-focused and content-focused e-collaborative writing tasks in Google Docs. *Education and Information Technologies*, 1-24. https://doi.org/10.1007/s10639-022-11039-y

Keshanchi, E., Pourdana, N., & Famil Khalili, G. (2022). Differential Impacts of pair and self-dynamics on written languaging attributes and translation task performance in EFL context. *English Teaching & Learning*, 1-22. https://doi.org/10.1007/s42321-022-00113-w

Lee, L., Gundersen, E., & Bernard, J. (2011). *Select readings*: *Intermediate.* Oxford University Press.

Lee, Y. W. (2015). Diagnosing diagnostic language assessment. *Language Testing, 23*(3), 299–316. https://doi.org/10.1177/0265532214565387

Lenhard, W., & Lenhard, A. (2016). *Calculation of effect sizes.* Psyhometrica, Bibergau.

Levis, J., & Pickering, L. (2004). Teaching intonation in discourse using speech visualization technology. *System, 32*, 505-524. https://doi.org/10.1016/j.system.2004.09.00

Levy, H. M. (2008). Meeting the needs of all students through differentiated instruction: Helping every child reach and exceed standards. Clearing House: A Journal of Educational Strategies, Issues, and Ideas, 81(4), 161–164. https://doi.org/10.3200/TCHS.81.4.161-164

Lindner, K., & Schwab, S. (2020): Differentiation and individualization in inclusive education: a systematic review and narrative synthesis. *International Journal of Inclusive Education.* https://doi.org/10.1080/13603116.2020.1813450

Nour, P., Esfandiari, R., & Zarei, A.A. (2021). Development and validation of a metamemory maturity questionnaire in the context of English as a foreign language. *Language Testing in Asia,* *11*, 24. doi:10.1186/s40468-021-00141-6

Nunley, K. F. (2006). *Differentiating the high school classroom: Solution strategies for 18 common obstacles*. Thousand Oaks.

Pallant, J., (2010). *SPSS survival manual: A step by step guide to data analysis using the SPSS program* (4^th^ ed.). McGraw Hill.

Parsons, S. A., Vaughn, M., Scales, R. Q., Gallagher, M. A., Parsons, A. W., Davis, S. G., . . . Allen, M. (2018). Teachers’ instructional adaptations: A research synthesis. *Review of Educational Research, 88*(2), 205–242. https://doi.org/10.3102/0034654317743198

Pham, H. L. (2012). Differentiated instruction and the need to integrate teaching and practice. *Journal of College Teaching & Learning, 9*(1), 13-20. https://doi.org/10.19030/tlc.v9i1.6710

Pourdana, N. (2022). Impacts of computer-assisted diagnostic assessment on sustainability of L2 learners’ collaborative writing improvement and their engagement modes. *Asian-Pacific Journal of Second and Foreign Language Education*, *7*(1), 1-21. https://doi.org/10.1186/s40862-022-00139-4

Pourdana, N., & Tavassoli, K. (2022). Differential impacts of e-portfolio assessment on language learners’ engagement modes and genre-based writing improvement. *Language Testing in Asia, 12*(7), 1-19. https://doi.org/10.1186/s40468-022-00156-7

Pourdana, N., & Asghari, S. (2021). Different dimensions of teacher and peer assessment of EFL learners’ writing: Descriptive and narrative genres in focus. *Language Testing in Asia, 11*(6). https://doi.org/10.1186/s40468-021-00122-9

Pourdana, N., & Mohamadi Zenouzagh, Z. (2021). Feedback types, negotiation of meaning, and negotiation of form in multimodal and text-based computer-mediated English collaborative writing. *Foreign Language Research Journal, 11*(3), 453-471. https://doi.org/10.22059/JFLR.2021.321348.825

Pourdana, N., Nour, P., & Yousefi, F. (2021). Investigating metalinguistic written corrective feedback focused on EFL learners’ discourse markers accuracy in mobile-mediated context. *Asian-Pacific Journal of Second and Foreign Language Education,
6*(7), https://doi.org/10.1186/s40862-021-00111-8.

Pourdana, N., & Rad, S. M. (2017). Differentiated instructions: Implementing tiered listening tasks in mixed-ability EFL context. J*ournal of Modern Research in English Language Studies, 4*(1), 87-69. https://doi.org/10.30479/JMRELS.2017.1566

Rafi, F., Pourdana, N., & Ghaemi, F. (2022). Computer-mediated diagnostic assessment of mixed-ability EFL learners’ performance on tiered tasks: Differentiating mediation on Google Meet™. *Journal of Modern Research in English Language Studies.* http://dx.doi.org/10.30479/jmrels.2021.16118.1950

Reid, D., & Feist, D. (2018). Questioning the role of tomorrow's teacher: Technology-facilitated differentiated learning in 21st-century classrooms. In *Handbook of Research on Pedagogical Models for Next-Generation Teaching and Learning* (pp. 135-153): IGI Global.

Ritter, O. N. (December 2018). *Integration of educational technology for the purposes of differentiated instruction in secondary STEM education.* Retrieved January 7, 2022, from https://trace.tennessee.edu/utk_graddiss/5274.

Seferoglu, G. (2005) Improving students’ pronunciation through accent reduction software. *British Journal of Educational Technology, 36*(2), 303–316. https://doi.org/10.1111/j.1467-8535.2005.00459

Scalise, K. (2007). Differentiated e-learning: Five approaches through instructional technology. *International Journal of Learning Technology, 3*(2), 169-182. https://doi.org/10.1504/IJLT.2007.014843

Shepherd, C. M., & Alpert, M. (2015). Using technology to provide differentiated instruction for deaf learners. *Journal of Instructional Pedagogies, 16*.

Storch, N. (2013). *Collaborative writing in L2 classrooms.* Multilingual Matters.

Suwastini, N. K. A., Rinawati, N. K. A., Jayantini, I., & Dantes, G. (2021). Differentiated instruction across EFL classrooms: A conceptual review. *TELL-US Journal, 7*(1), 14-41. https://doi.org/10.22202/tus.2021.v7i1.4719

Swain, M. (2001). Examining dialogue: Another approach to content specification and to validating inferences drawn from test scores. *Language Testing, 18*(3), 275–302. https://doi.org/10.1177/026553220101800302

Tabachnick, B. G. & Fidell, L. S. (2013). *Using multivariate statistics* *(6^th^ ed.)*. Pearson Education.

Tavassoli, K., Bashiri, L., & Pourdana, N. (2022). [Experienced and novice L2 raters’ cognitive processes while rating integrated and independent writing tasks](https://jle.hse.ru/article/view/13466). *Journal of Language and Education, 8*(4). 168-180. https://doi.org/10.17323/jle.2022.13466

Taylor, B. K. (2015). Content, process, and product: Modeling differentiated instruction. *Kappa Delta Pi Record, 51*(1), 13-17. http://dx.doi.org/10.1080/00228958.2015.988559

Tomlinson, C. A., Brighton, C., Hartberg, H., Callahan, C. M., Moon, T. R., Brimijoin, K., . . . Reynolds, T. (2003). Differentiating instruction in response to student readiness, interest, and learning profile in academically diverse classrooms: A review of the literature. *Journal for the Education of the Gifted, 27*(2-3), 119-145.

Tomlinson, C. (2014). *The differentiated classroom: Responding to the needs of all learners* (2nd ed.). ASCD.

Tozcu, A. (2016). The effectiveness of diagnostic assessment on the development of Turkish language learners’ narrative skills as an oral proficiency interview (OPI) task. *Journal of the National Council of Less Commonly Taught Languages, 19*(1), 61–96.

van Geel, M., Keuning, T., Frèrejean, J., Jeroen, D., van Merriënboer, J., & Visscher, J. A. (2019) Capturing the complexity of differentiated instruction. *School Effectiveness and School Improvement, 30*(1), 51-67. https://doi.org/10.1080/09243453.2018.1539013

Wang, X. & Munro, M. J. (2004). Computer-Based Training for Learning English Vowel Contrasts. *System: An International Journal of Educational Technology and Applied Linguistics, 32*(4), 539-552. https://doi.org/10.1016/j.system.2004.09.011

Yan-hong, M. (2013). *The research of cooperative learning effects on college students’ learning anxiety.* David Publishing

Vygotsky, L. S. (1987). Thinking and speech. In R. W. Rieber, & A. S. Carton (eds.), *The collected works of* *L. S. Vygotsky: Vol. 1: Problems of general psychology* (pp. 39-285). Plenum.

**Appendix A**

**Oral Tiered Tasks # 4 (Sample)**


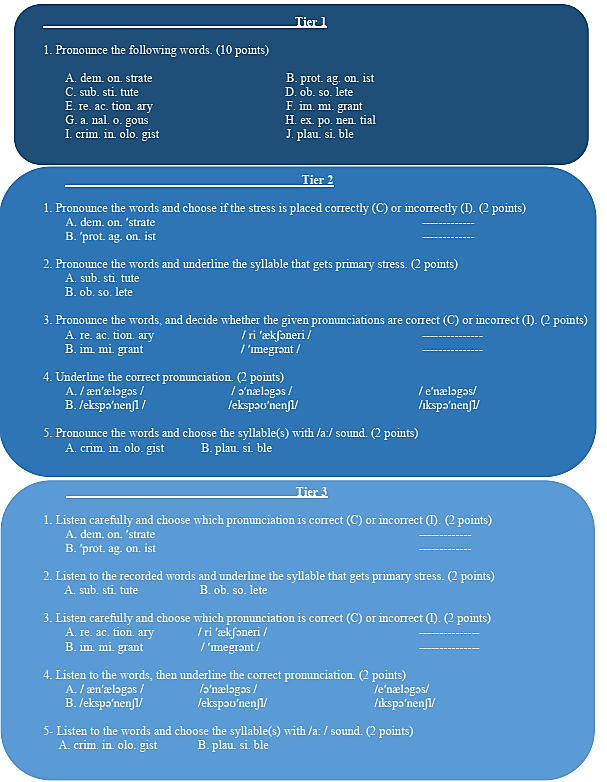

Supplement: Supplementary file 1 — Additional file 1: Appendix A. Oral-tiered tasks # 4 (sample). [file 40468_2023_223_MOESM1_ESM.docx]
